# Development of a TaqMan Array Card for Pneumococcal Serotyping on Isolates and Nasopharyngeal Samples

**DOI:** 10.1128/JCM.00613-16

**Published:** 2016-06-24

**Authors:** Suporn Pholwat, Fuminori Sakai, Paul Turner, Jorge E. Vidal, Eric R. Houpt

**Affiliations:** a Division of Infectious Diseases and International Health, Department of Medicine, University of Virginia, Charlottesville, Virginia, USA; b Hubert Department of Global Health, Rollins School of Public Health, Emory University, Atlanta, Georgia, USA; c Cambodia Oxford Medical Research Unit, Angkor Hospital for Children, Siem Reap, Cambodia; d Centre for Tropical Medicine and Global Health, Nuffield Department of Medicine, University of Oxford, Oxford, United Kingdom; Cleveland Clinic

## Abstract

Streptococcus pneumoniae is both a commensal and a major pathogen that causes invasive disease in people of all ages. The introduction of serotype-specific pneumococcal vaccines has reduced the burden of disease but has also led to replacement with new strains; thus, serotyping remains important for vaccine-related disease surveillance. Conventional serotyping methods are laborious and expensive. We developed an easy-to-perform genotypic TaqMan array card (TAC) to identify S. pneumoniae strains, including *lytA*-based sequences, and 53 sequence-specific PCRs to identify 74 serotypes/serogroups covering all current vaccine types as well as prevalent nonvaccine types. The TAC method was evaluated on 146 clinical S. pneumoniae isolates and 13 nonpneumococcal species that naturally inhabit the upper respiratory tract and yielded 97% (142/146) sensitivity and 100% (13/13) specificity versus results of standard Quellung serotyping. The calculated limit of detection was 20 to 200 fg (∼8 to 84 genome equivalents) per reaction. On 23 blinded nasopharyngeal specimens that were pneumococcus culture positive, the TAC pan-pneumococcus *lytA* assay was positive in 21 (91% sensitivity versus culture). On TAC *lytA*-positive specimens, a serotype result was obtained on 86%, and the result was 95% accurate versus the subsequent culture's Quellung result. TAC also detected mixed serotypes in two specimens where Quellung detected only the predominant serotype. This TAC method yields fast and comprehensive serotyping compared to the standard method and may be useful on direct specimens.

## INTRODUCTION

*S*treptococcus pneumoniae (the pneumococcus) is a leading invasive pathogen of children and older adults, principally causing pneumonia, otitis media, and meningitis. The precursor to invasive disease is upper airway colonization (1). Existing vaccines are based upon capsular polysaccharide and are highly effective only against vaccine types (2–5). Serotype replacement occurs with increased colonization and disease caused by nonvaccine strains (6, 7). With over 90 different capsular serotypes, there is a constant race to add more capsular types to further expand coverage to reduce disease burden amid a headwind of changing strain replacement.

In this context it is important to epidemiologically follow pneumococcal serotypes, both in invasive strains to detect emergence of virulent serotypes and also in the upper airway to monitor strain replacement (1, 8). However, serotyping of pneumococci with the Quellung method is technically difficult, requires expensive panels of polyclonal antisera and precise inocula (9), and may yield visually ambiguous reactions (10). Furthermore, a limited number of subcultured colonies are typed, limiting the ability to detect mixed infections, particularly from nasopharyngeal specimens (11, 12).

Molecular serotyping methods are therefore emerging. After elucidation of the capsular biosynthetic locus (13), PCR assays for the capsular polysaccharide synthesis gene clusters have been devised. Sequencing-based assays of the *cps* and *wzh* genes (14, 15) have been published, as have real-time PCR assays to detect 21 serotypes/serogroups (16, 17). Nanofluidic, microarray, and Luminex-based systems have also been developed (18–21). Recently, we optimized 53 singleplex reactions to discern most serotypes/serogroups, including all vaccine types (22). However, performing that many reactions per specimen is onerous and difficult to implement in field settings; therefore, in this work we further optimized and configured the reactions to a single TaqMan array card (TAC).

## MATERIALS AND METHODS

### Bacterial strains.

All bacterial strains utilized in this study were cultured at Emory University on blood agar plates and incubated at 37°C with 5% CO_2_ overnight (∼16 h) prior to DNA extraction. Strains from 70 S. pneumoniae included serotypes 1, 2, 3, 4, 5, 6A, 6B, 6C, 7A, 7B, 7F, 8, 9L, 9N, 9V, 10A, 10B, 10F, 11A, 11B, 11C, 11F, 12B, 12F, 13, 14, 15A, 15B, 16A, 16F, 17A, 17F, 18C, 19A, 19B, 19C, 19F, 19“F” (atypical), 20, 21, 22A, 22F, 23A, 23B, 23F, 24A, 24B, 25A, 27, 28A, 28F, 29, 31, 33A, 33B, 33D, 33F, 34, 35A, 35B, 35F, 36, 38, 39, 41A, 41F, 43, 45, 46, and 47A, as described previously (22). For specificity testing, we included 20 streptococci naturally found in the nasopharynx, including S. infantis, S. oralis, S. anginosus, S. intermedius, S. sobrinus, S. pseudopneumoniae, S. mitis, S. parasanguinis, S. australis, S. mutans, S. peroris, S. oligofermentans, S. intestinalis, S. vestibularis, S. cristatus, S. salivarius, S. gordonii, S. sanguinis, S. sinensis, Dolosigranulum pigrum, and three other bacterial species Neisseria meningitidis, Haemophilus influenzae, and Staphylococcus aureus.

### Nasopharyngeal samples from children.

Nasopharyngeal (NP) samples (*n* = 28) belonged to our laboratory collection, and pneumococcal carriage had been analyzed in our previous studies (10, 23). NP samples were stored at −80°C in skim milk-tryptone-glucose-glycerin (STGG) transport medium prior to DNA extraction.

### Quellung standard serotyping.

Quellung results were determined as described previously (22). Briefly, a fresh overnight bacterial culture in a blood agar plate was suspended in 1× phosphate-buffered saline (PBS) and then mixed with antiserum on a glass slide and read microscopically at a magnification of ×100. Pneumococcus Neufeld antiserum was obtained from the Statens Serum Institute (Copenhagen, Denmark).

### DNA extraction from bacterial cultures and nasopharyngeal specimens.

A bacterial colony was suspended in 200 μl of lysis buffer (Tris-EDTA [TE] buffer containing 0.04g/ml lysozyme and 75 U/ml mutanolysin), or 200 μl of nasopharyngeal specimens (in STGG medium) was mixed with 100 μl of lysis buffer. Samples were incubated for 1 h at 37°C. DNA was then purified using a QIAamp DNA minikit (Qiagen, Valencia, CA, USA) according to the manufacturer's instructions and eluted in 100 μl. The quality and quantification of DNA preps obtained from bacterial cultures were further evaluated using a NanoDrop system (NanoDrop Technologies, Wilmington, DE).

### Assay development on 384-well plates.

We adopted 53 serotype/serogroup-specific primers and probes from published sources (16, 22, 24–26) (Table 1) and, if needed, made modifications to accommodate the common cycling condition of the TaqMan array card (TAC) using Primer Express, version 3 (Applied Biosystems, Life Technologies Corp., Carlsbad, CA, USA). We also included one pan-pneumococcus assay (*lytA*) (26) and an assay for an internal control (27). Optimization of conditions and probe specificity testing were performed using the 384-well format of the ViiA7 platform (Applied Biosystems, Life Technologies Corp., Carlsbad, CA, USA). Each primer/probe set (0.09 μl of each forward and reverse primer, 0.025 μl of probe of a 50 μM stock) was amplified in singleplex in a total of 5 μl of PCR mixture containing 2.5 μl of 2× TaqMan universal master mix II with uracil-*N* glycosylase (UNG) (Applied Biosystems, Life Technologies Corp., Carlsbad, CA, USA), 1.295 μl of nuclease-free water, and 100 pg of genomic DNA. Cycling conditions included UNG activation at 50°C for 2 min and initial denaturation at 95°C for 10 min, followed by 40 cycles of denaturation at 95°C for 10 s and annealing/extension at 60°C for 1 min. We included 54 previously characterized serotypes in each run for specificity testing, and nuclease-free water was used for a nontemplate control.

**TABLE 1:** Primer and probe sequences of the 53 PCRs corresponding to 74 serotypes

Serotype or sample type	Target	Sequence (5′–3′)^*a*^	Reference(s) or source
1	*wchD*	F-CGTGCGGTAATTGAAGCTATGA	24
		R-TGTGGCCCCAGCAACTCT	
		P-TGCTTGCCCTTGTATAGGGT	
2	*wzy*	F-TTATGGACTGGCTGATGGTTCTC	25
		R-AAATCCTGACCCAATAATAGCCTTT	
		P-AGGTCAACGTATTGGAACTCTTAGAAATTGGGAAA	
3	*tnp*	F-GGTCAGCAGAAAGTATGCATTGG	22, 24
		R-TCGTTTATCCAGGGTCTGATGA	
		P-TATTGGATGTGGTTTATCGTGAAGA	
4	*wzy*	F-GCATCAGCGACGGTTGTTAT	This study
		R-CACCACCATAGTAACCAAAGTTCC	16
		P-TTACCTGTAGGCTCTTCTTTTG	16 (modified)
5	*wzy*	F-CATGATTTATGCCCTCTTGCAA	16 (modified)
		R-GACAGTATAAGAAAAAGCAAGGGCTAA	
		P-CTTCTTCTCATCGTTTCCGCAT	
6ABCD	*wciP*	F-AAGTTTGCRCTAGAGTATGGGAAGGT	22, 24 (modified)
		R-ACATTATGTCCATGTCTTCGATACAAG	
		P-TGTTCTGCCCTGAGCAACTGG	
6CD	*wciN*_beta_	F-CAATCAGGCAGTTCTTTTCTCG	22
		R-ACCTGACTCACCATCAATAACC	
		P-AAATGGGAGGGCTTTGGATTGGC	
7AF	*wcwH*	F-ATGAAGGCTTTGGTTTGACAGG	16 (modified)
		R-ATTCTCGCCATCAATTGCATATTC	
		P-TGAGACTAACGCACAGCCA	
7BC-40	*wcxU*	F-TCCAGATATAGTCATTCCCAATCAG	22 (modified)
		R-AAAGAAGGTAAATCCCATGATGAATT	
		P-TCCCTCATTATCGATTACTGACCCACCA	
8	*wzx*	F-CCACTCATCAGTTTCCCATATGTTT	22, 24
		R-TCAATAATTGAAGAAGCGAACGTT	
		P-TGATGGCAGATGGGTTGGGACGAG	
9AV	*wzx*	F-AGGTATCCTATATACTGCTTTAGG	16 (modified)
		R-CGAATCTGCCAATATCTGAAAG	
		P-ACACATTGACAACCGCTACA	
9LN	*wzx*	F-CGTGGAATTTTCTATACTGCAATAGG	22 (modified)
		R-CTACTGCTACGATACCATATTCTACAG	
		P-CAATTCTTAGCCGGATTCTCTC	
10A	*wcrD*	F-AGAGGCCCTAAGAAAAGATTCG	22 (modified)
		R-CCCAGTCATCCCCATCAATAAC	
		P-AGGTCATGGCTCAACAATT	
10B	*wcrD*	F-AAATATGAGATTGGTAAGGAATATTCTGG	22
		R-GTCTTTTCACTTAAACGAATTCCATTC	
		P-AACGGATTCCAATGCACTCGGTAACT	
11AD	*wchK*	F-CGGCCCAGCTACATTTATGG	This study
		R-TGATCATTCACATGCTCACCAA	
		P-AAATACCAATAGTTGTTCCGAGATTAAAGAAGT	
11F*	*wchK*	F-TGGTCCAGCTACTTTTATGGC	22
		R-TGATCATTCACATGCTCCCC	
		P-ACTCCAATAGTTGTTCCGAGGCAAAAGA	
12ABF-44-46	*mnaB*	F-GCACCCACGGGTAAATATTCTAC	16 (modified)
		R-CAACTAAGAACCAAGGATCCACAG	
		P-ATACAATGCCCACCAACACC	
12B	*wzx*	F-GGTTGCTGATCAAAAGGTCTATG	This study
		R-AGGTTCAAAGTAAGATTTTTAGCAA	This study
		P-AGATAAAAATCTTTCCAAATCATCAAAGTGA	22 (modified)
13	*wzy*	F-AGACTACCATTTTTTGATCAGTTAGATT	22
		R-CAGAAAACATATTTTGTTCATAAATCCATC	
		P-AAGCAGCACTTCCAAGTCGTAATCTACC	
14	*wchL*	F-CGACTGAAATGTCACTAGGAGAAGAT	22, 24 (modified)
		R-AATACAGTCCATCAATTACTGCAATACTC	
		P-TCATTCGTTTGCCAATACTTGATGGTCTC	
15	*wzx*	F-TTGAATCAGGTAGATTGATTTCTGCTA	22, 24
		R-CTCTAGGAATCAAATACTGAGTCCTAATGA	
		P-CTCCGGCTTTTGTCTTCTCTGT	
16F	*wzy*	F-TAATGTTATGACCTTGGTAATCTTCCC	16 (modified)
		R-TCCCAAAGGATAATCAATAACTTTTAGAAG	
		P-TCTTCCAAATGCTTAACCGC	
17F	*abp2*	F-GGAACTGTTGATCATCTTAGCGTA	This study
		R-TTTTGATCCCGTACTCGGAAG	This study
		P-TCTTCGTATGCTAGTTCTAAGAGAGCTACTGA	25 (modified)
18ABCF	*wzy*	F-TCGATGGCTAGAACAGATTTATGG	16 (modified)
		R-CCATTGTCCCTGTAAGACCATTG	
		P-TTGAATCAACCTATAATTTCGCCCC	
19A	*wzy*	F-GCTCATTGATATCCAATTCTGGAA	This study
		R-CATGGCTAAGTGCAAGATTATGAATC	
		P-AGCTCTTACTATTATAGTTGACCTCATTATTCT	
19F	*wzy*	F-CGGGGTCAAATATTCAGTGG	This study
		R-CACGAATGAGAACTCGAATAAAAG	16
		P-TTCGCACTGTCAATTCACCT	16 (modified)
19“F”^*b*^	*wzy*	F-GTCCTTAGTTCTGGTTATTCGGG	22
		R-GGATGAGGAACCGAATCGAAG	
		P-CCAGTTATGAAGGTGAGCTAACAGTGCG	
20	*wciL*	F-AAAGATACTGGCTGAGGAGCTATCTATT	22, 24
		R-AGTCAAAAGTACTCAACCATTCTGATATATTC	
		P-AGGATAAGGTCTACTTTGTGGGAGTTC	
21*	*wzy*	F-GGTTTAAATATCGCTCCGGGTAT	25
		R-CAAAAAAAGGGCTTGTAGACGAA	
		P-TGTGAATTGGACACGTTATGGAGC	
22AF	*wcwA*	F-TCTCTGAAATGGTTGTTGAAGGAA	22, 24 (modified)
		R-TCGCATCCGATAGTTCTTGTGA	
		P-TGGCAATCCCAGGACAA	
23A	*wzy*	F-CTCCCCTCCATTACCCATTTGG	16 (modified)
		R-TGAAGAAAGTGCTGTTTGTGAACC	
		P-TCCCACACTCCCTACTCCCA	
23B	*wzx*	F-TTGAAGAAATTGCTCCAGAAACAT	22, 25
		R-CCAAAAGACTAGCCTCAACCACTAA	
		P-TAGAGCTATTTATCTTTCGTGGTTTT	
23F	*wzy*	F-AAGTGATAGTGAACTTGGGATTGTCT	This study
		R-GATTCTATTTGCAAACACGTTGAGA	
		P-TGTTAAAAATACACACAACATCAACA	
24A	*wzx*	F-CTTGGAGTTGCTAATTATGGGAAG	22 (modified)
		R-ATCTCTTACACGTGCACACTC	
		P-CACAGCATATCGTAAAATACCCGCA	
25AF*	*wcyE*	F-ATACCAACTAGAATCAGCAGGAC	22 (modified)
		R-AAATGGAATATCTTTTGATAATTTACTCGC	
		P-CCGCTGGACTTACTGCAATA	
27	*whaK*	F-AGCGATTTAGCGACTGATATCC	22
		R-TCTCAAAATCGATCTCGCGTG	
		P-TGTGGAAGGCGTTTGAAGGTGACT	
29*	*wcrJ*	F-TTCGAGTTGTGCCGTTTTTACA	25 (modified)
		R-GGCGTACCCACCTCTAAAATTTT	
		P-TGAATCCTAGTCTTTTCTCTGCG	
31	*wzy*	F-GCAGAAGTTTTAAGTCACGGAC	22
		R-AGCATTACAGATGTCACTAAGGG	
		P-CCCCCACGTAAAACCGCAAGG	
33AF-37	*wzy*	F-GGAACTGGTTCAGCAACTATACG	16 (modified)
		R-GGTTCTAAGACCGTCTGAAATACC	
		P-TAGGACTTTTCTGCCATGCC	
33B	*wciN*	F-CCTGTTAGTGCACCTGTATTTAAC	22 (modified)
		R-GCATTCAAAACTCCTTCATCTCC	
		P-TTCGTTGTTCACGCCATTTA	
33D*	*wciN*	F-CGTATAGTCTTGCGACATTTCA	22 (modified)
		R-TTCCACATGCGTTACCTCAC	
		P-CACAACTAGTTTTTTATCAAAAAGACCTTGGC	
34	*wzy*	F-CGGTGGAGTAGGTCAAGATG	22
		R-GTCTGTTCTCCCCAATATACTGAG	
		P-ACGGAGCGCCAATGTACTTGAATAGTT	
35AC-42	*wcrK*	F-TGTTTCAAGCTTCCCCTTTAGA	This study
		R-AAATGAAATCAAAGTATCACGTATCG	22 (modified)
		P-TTCAAAATACCCAGGACACCCGTTCA	22
35B	*wcrJ*	F-GCATGGAGGTGGAGCATACA	22, 24 (modified)
		R-TGTAAAGACTGCACAACTCGATATAAAA	
		P-AACAATATTAGTAAAGCGCAGGTC	
35F-47F	*wzy*	F-GTGGTCGTATATACTTGATGAATAAATCG	22 (modified)
		R-ACATACAAATTATCAACATACAGATAGGTC	
		P-TTCAACTGGTCGTCCGAATA	
36*	*wzy*	F-CTTGTCTATTCAGCCCTTCTGG	22 (modified)
		R-CGCGATTATATTGTAAATTGGGAACT	
		P-AGAATGCCCGCTACAATGAG	
38-25AF	*wciI*	F-GTCTTACGTAGAACCTCTCTGGATGA	22, 24
		R-TGGTCCTACAAGCGACATGTG	
		P-TTGCCACAGATTTGGAATATTTTGGTCGG	
39*	*wcrG*	F-CAAAAAAATGAACTAACTCAAATAGTAACG	22
		R-ATACTGTAATTTTCTTGTTTATTTGCGG	
		P-AAGTCAGGCGTATTCTTCACAAGGGAAA	
41A	*wciB*	F-GCAAATAGATGTATCCCAGTTAACAC	22 (modified)
		R-GGTAGCTCTTTTGGTTTAATGTCC	
		P-CGACCGAATAGTCTAGCTTCAAAGG	
41F	*wzx*	F-TTTTTGGGAGGAAGTGCTTTT	22
		R-AACCGCTTTCTCATGATTCATAACT	This study
		P-CTTCTGTGCTAACAGTGGAGAT	22 (modified)
43	*wzx*	F-AGAGGCTACATCAAATAGTTGGC	22
		R-GAATCACACCGTAACTTCCAAAG	
		P-TCCAATAGTACTCACCCCTACCGAGC	
45*	*wzy*	F-TCTAGCTACTTGACTAAAATATTTGAACTG	22 (modified)
		R-GACGAGTCGATTTCGCTGTAT	
		P-CTTTTAGTGACCTCGCTCCC	
47AF*	*whaI*	F-AGGAATTGGTAGAGAGTTTGTGG	22
		R-GAAAGTTGAACCATCATCCGTC	
		P-CACTTGATGGAATGCCTGCTGCC	
*lytA*	*lytA*	F-TCGTGCGTTTTAATTCCAGCT	26 (modified)
		R-ACGCAATCTAGCAGATGAAGCA	
		P-CTCCCTGTATCAAGCGTTTTCGGCA	
PhHV	*gB*	F-GGGCGAATCACAGATTGAATC	27
		R-GCGGTTCCAAACGTACCAA	
		P-TATGTGTCCGCCACCATCT	

a F, forward primer; R, reverse primer; P, probe labeled with FAM (6-carboxyfluorescein) except for the probes for the serotypes marked with asterisks, which are labeled with VIC at the 5′ end. All are 3′ minor groove binder probes.

b Atypical 19F.

### Evaluation of the TaqMan array card.

**FIG 1:**
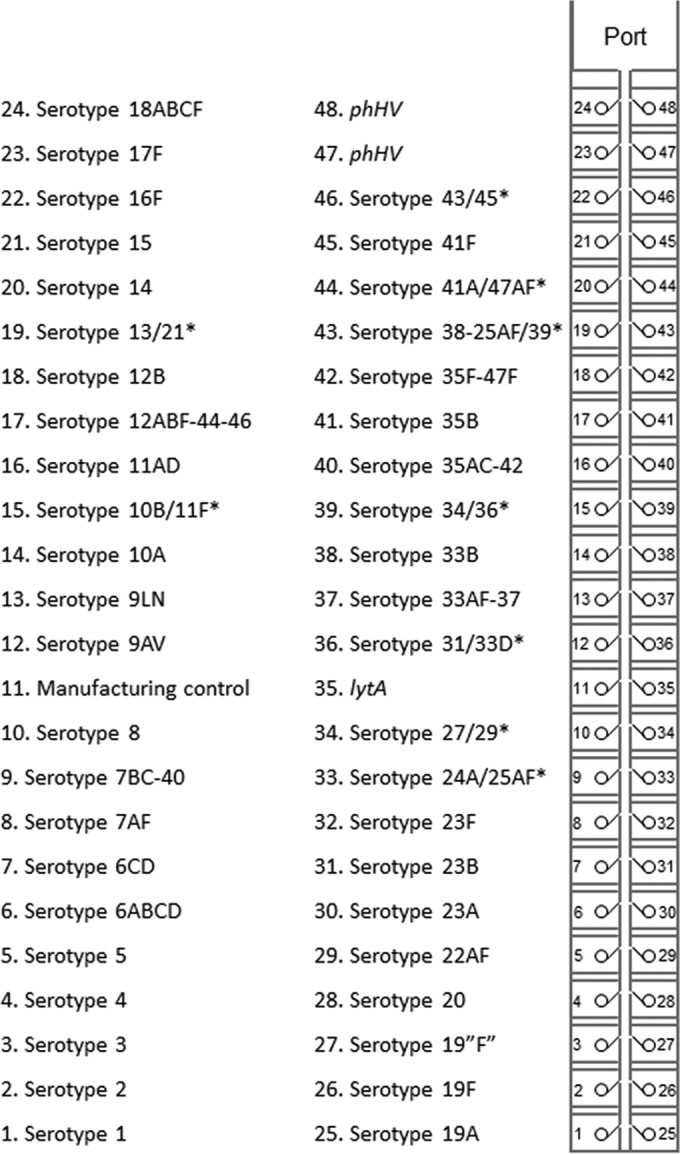
Streptococcus pneumoniae serotyping TaqMan array card layout. The TaqMan array card includes eight sample ports, whereby each sample is aliquoted into 48 PCRs. Serotypes in the form AB or A-B indicate a common assay that detects multiple serotypes/serogroups. Serotypes in the form A/B* indicate a duplex assay.

Primer and probe oligonucleotides were synthesized and spotted onto the TaqMan array card by Applied Biosystems (Life Technologies Corp., Carlsbad, CA, USA) as laid out in Fig. 1. Twenty microliters of input DNA (1 ng/μl for isolates) was mixed with 50 μl of 2× TaqMan universal master mix II with UNG (Applied Biosystems, Life Technologies Corp., Carlsbad, CA, USA) and 30 μl of nuclease-free water to a 100-μl final volume. This was loaded into each port of the card, whereby each card included seven clinical samples and one synthetic positive-control plasmid (Genewiz, Inc., South Plainfield, NJ, USA) that we designed to contain the primer and probe region of all 55 assays (53 serotype-specific assays plus *lytA* and phocine herpesvirus [PhHV]). The loaded card was centrifuged twice at 1,200 rpm for 1 min and then sealed; the loading ports were excised, and the card was inserted into a ViiA7 instrument (Life Technologies Corp., Carlsbad, CA, USA) and run under the same cycling conditions as described above for 40 cycles.

### Statistical analysis.

Means or medians were compared using Student's *t* test or a Mann-Whitney test. Data are shown as means ± standard deviations unless otherwise stated. A standard curve of *lytA* was generated with known DNA concentrations and plotted against the threshold cycle (*C_T_*) to yield the copy number, calculated as 10^(CT − 33.701)/−3.4262^.

## RESULTS

### Analytical assay performance.

The 53 serotype/serogroup-specific singleplex PCR assays as well as the S. pneumoniae lytA assay were tested against 54 serotyped pneumococcal isolates as well as several nonpneumococcal species, and 100% specificity with no cross-reactivity was observed (see Fig. 1 in the supplemental material). The PCR performance of each primer/probe assay was determined on both 384-well plates and the TaqMan array card formats. DNA from both individual and pooled serotypes was tested in serial dilution. The overall linearity of the 53 serotype assay targets including *lytA* was 0.997 ± 0.01 and 0.986 ± 0.02, and the PCR efficiencies were 93% ± 4.9% and 97% ± 9.7% using the 384-well plates and TaqMan array card formats, respectively. The limit of detection on 384-well plates was 10 to 100 fg/reaction while that of TAC was 20 to 200 fg/reaction (Table 2).

**TABLE 2:** PCR performance of each serotype/serogroup-specific assay

Serotype or sample	384-well plate		TaqMan array card	
	Linearity (*R*^2^)^*a*^	LOD (fg)^*b*^	Linearity (*R*^2^)	LOD (fg)
1	0.953 (87.2)	10 (4.2)	0.994 (85.7)	20 (8.4)
2	0.998 (85.9)	10 (4.2)	0.940 (87.3)	20 (8.4)
3	0.999 (97.7)	10 (4.2)	0.998 (98.8)	20 (8.4)
4	0.998 (90.0)	100 (42)	0.998 (95.6)	200 (84)
5	0.997 (89.9)	10 (4.2)	1.000 (92.8)	20 (8.4)
6ABCD	0.999 (92.4)	10 (4.2)	0.995 (98.9)	20 (8.4)
6CD	0.999 (91)	10 (4.2)	0.998 (98.6)	20 (8.4)
7AF	1.000 (92.9)	10 (4.2)	0.998 (89.5)	20 (8.4)
7BC-40	0.999 (91.1)	10 (4.2)	0.968 (80.4)	20 (8.4)
8	0.999 (100)	10 (4.2)	0.996 (99.0)	20 (8.4)
9AV	0.992 (95.7)	10 (4.2)	0.993 (109.1)	20 (8.4)
9LN	0.993 (90.6)	10 (4.2)	0.957 (87.4)	20 (8.4)
10A	1.000 (97.0)	10 (4.2)	0.983 (102.6)	20 (8.4)
10B	1.000 (94.4)	10 (4.2)	0.996 (108.1)	20 (8.4)
11AD	1.000 (92.4)	10 (4.2)	0.965 (86.5)	20 (8.4)
11F	0.994 (84.8)	10 (4.2)	0.943 (120.2)	20 (8.4)
12ABF-44-46	0.999 (99.2)	10 (4.2)	0.997 (102.3)	20 (8.4)
12B	1.000 (91.5)	100 (42)	0.961 (76.1)	200 (84)
13	0.999 (90.9)	100 (42)	0.861 (94.8)	200 (84)
14	0.999 (95.7	10 (4.2)	0.994 (95.7)	20 (8.4)
15	1.000 (93.7)	10 (4.2)	0.994 (102.6)	20 (8.4)
16F	0.998 (86.9)	100 (42)	0.979 (92.5)	200 (84)
17F	0.998 (88.5)	10 (4.2)	0.996 (103.3)	20 (8.4)
18ABCF	0.999 (91.0)	10 (4.2)	0.998 (78.4)	20 (8.4)
19A	1.000 (92.8)	10 (4.2)	0.994 (99.5)	20 (8.4)
19F	0.999 (99.3)	10 (4.2)	0.998 (95.0)	20 (8.4)
19“F”	1.000 (94.4)	10 (4.2)	0.993 (94.4)	20 (8.4)
20	0.998 (95.3)	10 (4.2)	0.996 (90.6)	20 (8.4)
21	0.999 (93.4)	10 (4.2)	0.993 (98.2)	20 (8.4)
22AF	0.998 (100)	10 (4.2)	0.999 (103.5)	20 (8.4)
23A	0.999 (89.3)	10 (4.2)	0.995 (92.8)	20 (8.4)
23B	0.998 (103)	10 (4.2)	0.992 (103.7)	20 (8.4)
23F	0.996 (88.5)	10 (4.2)	0.998 (93.6)	20 (8.4)
24A	0.998 (100)	10 (4.2)	0.973 (82.8)	20 (8.4)
25AF	0.997 (88.5)	10 (4.2)	0.997 (103.6)	20 (8.4)
27	0.998 (89.9)	10 (4.2)	0.996 (106.7)	20 (8.4)
29	0.992 (85.5)	10 (4.2)	0.997 (70.0)	20 (8.4)
31	0.999 (93.1)	10 (4.2)	0.996 (89.7)	20 (8.4)
33AF-37	1.000 (90.7)	10 (4.2)	0.998 (101.3)	20 (8.4)
33B	0.993 (97.8)	10 (4.2)	0.996 (98.9)	20 (8.4)
33D	1.000 (96.5)	10 (4.2)	0.985 (95.1)	20 (8.4)
34	0.996 (92.6)	100 (42)	1.000 (103.4)	200 (84)
35AC-42	0.997 (105)	10 (4.2)	0.996 (103.8)	20 (8.4)
35B	0.998 (94.0)	10 (4.2)	0.979 (100.2)	20 (8.4)
35F-47F	0.997 (90.1)	10 (4.2)	0.996 (94.3)	20 (8.4)
36	0.999 (88.7)	10 (4.2)	0.997 (103.1)	20 (8.4)
38-25AF	0.999 (96.8)	10 (4.2)	1.000 (94.3)	20 (8.4)
39	0.999 (96.4)	10 (4.2)	0.986 (108.8)	20 (8.4)
41A	1.000 (91.7)	10 (4.2)	0.994 (97.9)	20 (8.4)
41F	1.000 (79.0)	100 (42)	0.997 (105.7)	200 (84)
43	0.998 (95.4)	10 (4.2)	0.929 (96.8)	20 (8.4)
45	1.000 (94.7)	10 (4.2)	0.998 (95.0)	20 (8.4)
47AF	1.000 (93.4)	100 (42)	0.995 (104.3)	200 (84)
*lytA*	0.998 (94.8)	10 (4.2)	0.999 (97.9)	20 (8.4)
PhHV	0.994 (94.7)		0.986 (114.9)	

a Values in parentheses represent PCR efficiency (%).

b LOD, limit of detection. Values in parentheses are the numbers of copies per reaction. The genome size of S. pneumoniae serotype 4 TIGR4 (2,160,842 bp) was used for calculations.

### Evaluation of TaqMan array card serotyping on clinical isolates.

The performance of the TaqMan array card was evaluated on 54 S. pneumoniae serotypes (previously serotyped by Quellung reactions) and then on 92 blinded isolates, which included serotypes 1, 2, 3, 4, 5, 6A, 6B, 7F, 8, 9N, 9V, 10A, 10F, 11A, 11B, 11C, 12F, 14, 15B, 16A, 17A, 17F, 18C, 19A, 19B, 19C, 19F, 20, 22A, 22F, 23F, 24B, 28A, 28F, 33F, and 13 non-S. pneumoniae strains. TAC yielded 97% (155/159) agreement compared with the Quellung serotype result (Table 3). There was 100% specificity, and all four discordant samples were indicated as serotype 22F by Quellung (performed twice) but negative with the TAC 22AF assay. This was not due to 22AF assay failure since it was positive for three serotype 22A strains.

**TABLE 3:** Performance of TaqMan array card serotyping on isolates versus the Quellung standard

Isolate condition and Quellung serotype or sample type	No. of isolates tested	No. of concordant results	No. of discordant results	% Accuracy
Unblinded				
All serotypes from Table 2	54	54	0	100
Blinded^*a*^				
**1**	4	4	0	100
2	2	2	0	100
**3**	3	3	0	100
**4**	4	4	0	100
**5**	2	2	0	100
**6A**	3	3	0	100
**6B**	5	5	0	100
**7F**	4	4	0	100
8	3	3	0	100
9N	4	4	0	100
**9V**	4	4	0	100
10A	3	3	0	100
11A	3	3	0	100
12F	1	1	0	100
**14**	4	4	0	100
15B	2	2	0	100
17F	3	3	0	100
**18C**	4	4	0	100
**19A**	3	3	0	100
19F	5	5	0	100
20	3	3	0	100
22A	2	2	0	100
22F	4	0	4^*b*^	0
**23F**	4	4	0	100
33F	3	3	0	100
Serotypes not included in Table 2	10	10^*b*^	0	100
Nonpneumococcal bacteria	13	13^*c*^	0	100
Total	159	155	4	97

a Serotypes in bold are those included in PCV13.

b Positive with *lytA* but negative with any serotype-specific probe on the TAC.

c Negative with any probe on the TAC, including *lytA*.

### TaqMan array card serotyping on direct specimens.

Twenty-eight nasopharyngeal specimens underwent DNA extraction and TAC testing, and the results were compared with the culture and Quellung results (22). In 23 of these nasopharyngeal specimens an S. pneumoniae strain had been isolated whereas five were culture negative. The TAC yielded a *lytA*-positive result in 21/23 culture-positive specimens and was positive in 1/5 culture-negative specimens (*C_T_* of 34), corresponding to a TAC *lytA* sensitivity of 91% and specificity of 80% versus culture (Table 4). The serotype assays were exclusively positive in *lytA*-positive specimens (TAC serotype assays, 100% specificity versus *lytA*). In the 22 *lytA*-positive specimens, a serotype result was obtained for 19 specimens (TAC serotype assays, 86% sensitivity versus *lytA*). The TAC serotype result matched the Quellung result in 18 specimens (95% accuracy), the exception being one pneumococcus specimen nontypeable by Quellung that was serotype 4 by TAC (with a somewhat late *C_T_* of 30 compared with the other serotype 4 strains). There were two culture-positive specimens undetectable by *lytA* on the TAC, one of which was *lytA*-positive when larger volumes of DNA were used (1 μl; *C_T_* of 37) in a plate format and one of which remained negative. Likewise, we retested the three *lytA*-positive/serotype-negative samples with the serotype assays with larger volumes of DNA, and one became positive (1 μl; *C_T_* of 36). We used the TAC *lytA C_T_* to extrapolate the pneumococcal concentration in the nasopharyngeal specimens, and the two discrepant results (e.g., *lytA* positive/serotype negative) were both at the lowest concentration of 3 × 10^3^ to 4 × 10^3^ CFU/ml.

**TABLE 4:** Performance of TaqMan array card serotyping method on nasopharyngeal specimens

Quellung serotype or culture result^*a*^	TaqMan array card			
	*lytA C_T_*	Serotype result(s) (*C_T_*)	DNA (copies/reaction)	Predicted bacterial load from *lytA C_T_* (CFU/ml)
4	27	4 (29), 23B (30)	9.33E1	9.33E5
4	28	4 (28)	4.70E1	4.70E5
6A	27	6ABCD (29)	6.77E1	6.77E5
6A	Negative	Negative	NA^*b*^	NA
10A	31	10A (33)	6.49E0	6.49E4
11A	23	11AD (23)	4.96E2	4.96E6
11A	28	11AD (28)	5.94E1	5.94E5
11A	32	11AD (35)	4.16E0	4.16E4
11A	34	11AD (34)	8.59E−1	8.59E3
17F	30	17F (30)	1.32E1	1.32E5
17F	33	17F (36)	1.66E0	1.66E4
19A	29	19A (31), 6CD (33)	1.79E1	1.79E5
19A	28	19A (30)	3.71E1	3.71E5
19F	29	19F (29)	2.85E1	2.85E5
19F	28	19“F” (30)	4.20E1	4.20E5
19F	30	19F (30)	1.41E1	1.41E5
19F	28	19F (28)	5.04E1	5.04E5
19F	35	Negative	3.86E−1	3.86E3
23F	28	23F (31)	3.63E1	3.63E5
23F	27	23F (28)	7.45E1	7.45E5
23F	Negative	Negative	NA	NA
35F	35	Negative	3.16E−1	3.16E3
NT	27	4 (30)	3.08E1	3.08E5
Pneumococcal culture negative				
With positive TAC result (*n* = 1)	34	Negative	9.62E−1	9.62E3
With negative TAC result (*n* = 4)	Negative	Negative	NA	NA

a The Quellung reaction was performed with pure culture colonies from the same nasopharyngeal samples. NT, not typeable.

b NA, not applicable.

## DISCUSSION

In this work we describe the development of a TaqMan array card that compartmentalizes 53 reactions to detect 74 pneumococcal serotypes and that can be used on isolates or nasopharyngeal specimens. Once developed, the TAC assay is as simple to perform as a single PCR. The TAC assays exhibited excellent linearity and limits of detection, albeit they were slightly less sensitive than the assays in a plate format, where more DNA template can be added. This slight sensitivity loss may not be clinically deleterious, and certainly the procedural advantage of the TAC versus setting up 54 singleplex PCRs is enormous.

For isolates where abundant DNA is available, performance remained excellent, with 97% accuracy versus the Quellung result. Indeed, the card had 100% accuracy on blinded isolates from a wide variety of 24 serotypes, including all of the PCV13 strains. Discrepancies were observed only with 22F strains. Curiously, *wcwA* sequences, according to GenBank accession numbers CR931681.1 and CR931682.1, are identical between 22A and 22F in the primer region, yet the 22F strains did not amplify, while 22A did, suggesting that there is a disconnect between the available 22F GenBank sequence and these four strains (see Fig. 2 in the supplemental material). Future iterations of the assays can attempt to understand and improve this. This caveat aside, the assay is clearly robust for use on cultured isolates from invasive sites and should provide an important tool to document whether serotype replacement is occurring in invasive strains (28).

The assay also worked well on direct nasopharyngeal specimens, with a 91% sensitivity versus culture and an 86% accuracy of the serotype result versus the Quellung reaction on *lytA*-positive specimens. A few samples had low levels of DNA at the *lytA* or serotype level that could be rescued with larger amounts of DNA. Thus, the serotype result was 100% accurate for any *lytA* result of a *C_T_* of 34 or below (corresponding to a nasopharyngeal density of 8 × 10^3^ CFU/ml), which is how we would propose using the assay. This assay is suitable for monitoring pneumococcus density and mixed infections in nasopharyngeal specimens, which is of great interest in the effort to better document the phenomenon of serotype replacement in the nasopharynx after vaccination (6). Regarding mixed infections, there was one discrepant nasopharyngeal specimen which was nontypeable by Quellung but serotype 4 by TAC, which we hypothesize was mixed. It is also plausible that this specimen represents a weakly expressing strain. We think that applying TAC to nasopharyngeal colonization will be particularly useful to monitor vaccine effectiveness in communities over time, ensuring that vaccine types are being eliminated as expected. Nasopharyngeal materials are much easier to obtain than invasive isolates, particularly in children and in resource-limited settings where the burden is highest. It has been proposed that the absence of a vaccine type in nasopharyngeal specimens in children with pneumonia could be used as a surrogate for vaccine effectiveness (11).

Limitations of our study are that the number of direct specimens with culture- and serotype-confirmed results was small; thus, the sensitivity and specificity estimates of the TAC assay are approximate, and additional evaluation will be beneficial. Other investigators have found that nonpneumococcal streptococcal species can interfere with serotyping assays (29), so more direct specimen testing is needed. Although the limit of detection by TAC was within the range of other reported assays (26), it was 2-fold higher than that of the regular real-time PCR format (22). Some serotypes could not be identified individually by single assay sets. For example, to infer serotype 6AB we must detect serotypes 6ABCD in the absence of serotypes 6CD (Fig. 1). We certainly suspect that the serotype reactions may need to be modified over time to include alternate types.

We embarked on this project because we have demonstrated excellent performance and reproducibility of the TAC platform in multisite field studies in Africa and Asia (30), areas of high pneumococcal carriage, coinfection, and variable serotype distributions. While the real-time PCR instrument is costly (∼$75,000), it also performs routine real-time PCR. To our knowledge, the TAC platform exists in at least 13 countries across sub-Saharan Africa and South Asia. The TAC cards are stable at 4°C for at least 2 years and cost about $50 per specimen, or approximately $1 per reaction, which compares favorably with conventional Quellung testing, which can cost up to $100 per colony (12). In conclusion, the TaqMan array card is a fast, high-throughput, serotyping method for pneumococci that is suitable to field studies.

## Supplementary Material

Supplemental material
