# Supplementary material for: Development of a TaqMan Array Card for Pneumococcal Serotyping on Isolates and Nasopharyngeal Samples
Source: J Clin Microbiol. 2016 Jun 24;54(7):1842–50. doi: 10.1128/JCM.00613-16 (PMC4922116; doi:10.1128/JCM.00613-16)
Supplement: Supplemental material [file supp_54_7_1842__index.html]

Supplemental material 

# Development of a TaqMan Array Card for Pneumococcal Serotyping on Isolates and Nasopharyngeal Samples

## Supplemental material

- Supplemental file 1 -

  Fig. S1 (Specificity testing of 53 serotype/serogroup-specific assays plus *lytA*)

  PDF, 1.6M
- Supplemental file 2 -

  Fig. S2 (Serotype 22F-specific assay)

  PDF, 1.6M
