# Supplementary figures and images for: Development of a TaqMan Array Card for Pneumococcal Serotyping on Isolates and Nasopharyngeal Samples
Source: J Clin Microbiol. 2016 Jun 24;54(7):1842–50. doi: 10.1128/JCM.00613-16 (PMC4922116; doi:10.1128/JCM.00613-16)

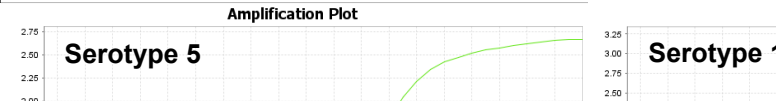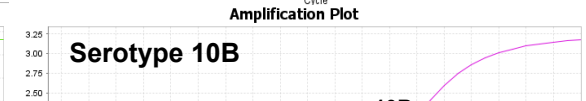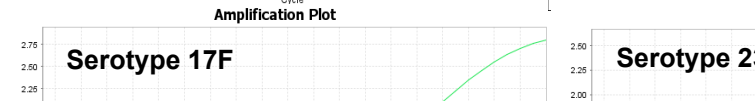

Supplement: Supplemental material [file JCM.00613-16_zjm999095054so1.pdf]
