# Supplementary material for: Development of a TaqMan Array Card for Pneumococcal Serotyping on Isolates and Nasopharyngeal Samples
Source: J Clin Microbiol. 2016 Jun 24;54(7):1842–50. doi: 10.1128/JCM.00613-16 (PMC4922116; doi:10.1128/JCM.00613-16)
Supplement: Supplemental material [file JCM.00613-16_zjm999095054so2.pdf]

A

```
                          *       520         *       540         *       560  Forward primer (Seq.)    *       600
22agi|6864 : AGAGGGTCAGGTTCAGGTTATCTATAACGGAGTGGACAATGCGATCTATCATCCGATGCAGTCAAGTGCAGTGCGTGAAAAATTTGGGATCCCAGAGGAT :  600
22fgi|6864 : .................................................................................................... :  600
7agi|68642 : ...A........A......G.A....................TG..........C.....AG....CA...........C......CT..T.....A..A :  600
7fgi|68642 : ...A........A......G.A....................TG..........C.....AG....CA...........C......CT..T.....A..A :  600
21gi|68643 : ...A..G.....C..............T...............G...............CAG....CA...........C.....TCT.....G..A..G :  600

                          *       620         *       640         *       660         *       680         *       700
22agi|6864 : GCTCTCGTCATTGGTATGGTTGGACGTGTCAACGCGTGGAAGGGGCAAGGAGATTTTCTAGAAGCTGTGACACCGATTTTGGAGCATAATCCGAATTCCG :  700
22fgi|6864 : .................................................................................................... :  700
7agi|68642 : T....T..............A..C..C........T........A..G.................A...G.G..............G.....C.....TA :  700
7fgi|68642 : T....T..............A..C..C........T........A..G.................A...G.G..............G.....C.....TA :  700
21gi|68643 : .....T.................T..C........T.............................G.....G..............G.....C...G..A :  700

                          *       720         *       740         *       760         *       780         *       800
22agi|6864 : TAGCTTTTCTAGCGGGGAGTGCCTTTGCTGGTGAAGAGTGGCGAGTTGAAGAATTAGAGTCTAAGATTTCCAAATCAAGTGTAGCCTCACAAATCAAACG :  800
22fgi|6864 : .................................................................................................... :  800
7agi|68642 : .C........G..A..A...........G..A.....................C.C..A....C....G............................... :  800
7fgi|68642 : .C........G..A..A...........G..A.....................C.C..A....C....G............................... :  800
21gi|68643 : .C........G..T..A....................................C.C............G...............T............... :  800

                          *       820         *       840         *       860         *       880         *       900
22agi|6864 : AATTGAATATTATGAGCATACGGCTGAACTTTATAATATGTTTGATATCTTTGTCTTACCAAGTACCAATCCCGATCCACTACCAACAGTGGTACTAGAG :  900
22fgi|6864 : ......................A............................................................................. :  900
7agi|68642 : ......C...............A....G.............................G.....C..............C.....G............... :  900
7fgi|68642 : ......C...............A....G.............................G.....C..............C.....G............... :  900
21gi|68643 : ......C...............A....G..........................T.................T.....C..................... :  900

                          *       920         *       940         *   Forward primer      *       980    Probe     1000
22agi|6864 : GCTATGGCTTGTGGTAAGCCAGTAGTCGGCTACCGTCATGGTGGTGTCTCTGAAATGGTTGTTGAAGGAACCAATGGCTTACTGGCAATCCCAGGACAAT : 1000
22fgi|6864 : .................................................................................................... : 1000
7agi|68642 : ..G.....A.....C..A........T.....T..G.............G.......A..AAG..G..C.AA..C..TC.C..A..C.CT...AATA.GC : 1000
7fgi|68642 : ..G.....A.....C..A........T.....T..G.............G.......A..AAG..G..C.AA..C..TC.C..A..C.CT...AATA.GC : 1000
21gi|68643 : ..G.....A..C..C..A..............T..............T.GC..........A......G..A..............C.CT...AAT..GC : 1000

              Reverse primer  1020         *      1040         *      1060         *      1080   Reverse primer (Seq.)
22agi|6864 : CACAAGAACTATCGGATGCGATTTTGGAGTTGGTTTCTGATCCAGAAAAACGGCTCCAATTTGGCCAAGCATCAGTAAGAAGACAAGGAGAATCGTTTTC : 1100
22fgi|6864 : .................................................................................................... : 1100
7agi|68642 : .TGC....T.G..TA.G..T...CAA..A....C.GACA..ATC..G...A.AGAA..G..A..AAGT.....TT.CCA.C.T...AA.....TC..... : 1100
7fgi|68642 : .TGC....T.G..TA.G..T...CAA..A....C.GACA..ATC..G...A.AGAA..G..A..AAGT.....TT.CCA.C.T...AA.....TC..... : 1100
21gi|68643 : .TGCT.........A.A.TC...CAA..A.....GGAGA.......G...A.AAA.....................GA........A....G.T...... : 1100

                          *      1120         *      1140         *
22agi|6864 : TTTGGAATCTTATATCCGTAGTTTCTCAGAATTATATAAGAGTTATAAAAATGAATAA : 1158
22fgi|6864 : .......................................................... : 1158
7agi|68642 : G......AG......T..G.A.........GC.......A.AA..CT..--------- : 1149
7fgi|68642 : G......AG......T..G.A.........GC.......A.AA..CT..--------- : 1149
21gi|68643 : ...A..TAG...C..TAAG.AC.....G..G..G.....A.CAG...G...A..T..G : 1158
```

B

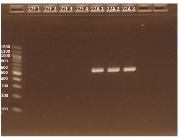

Supplemental Figure 2. Serotype 22F assay. The *wcwA* sequence alignment of serotype 7A, 7F, 21, 22A, and 22F is shown. Identical sequence with the top sequence (22A) are indicated by ".", different sequences are indicated by the nucleotide symbol (A, T, C, G), and "-" indicates no sequence available. The serotype 22AF specific primers (blue) and probe (green) are highlighted (A). Amplification of a larger 22AF sequence specific region of *wcwA* (550 bp, using the red primers) from DNA of 7 strains showed amplification in all 22A strains but no 22F strains (B).
